# Psychometric Properties of a Moroccan Version of the Summary of Diabetes Self-Care Activities Measure

**DOI:** 10.1155/2016/5479216

**Published:** 2016-02-25

**Authors:** Latifa Adarmouch, Majda Sebbani, Abdelhadi Elyacoubi, Mohamed Amine

**Affiliations:** ^1^Clinical Research Unit, Mohammed VI University Hospital, 40000 Marrakech, Morocco; ^2^Community Medicine and Public Health Department, School of Medicine, Cadi Ayyad University, Sidi Abbad, 40000 Marrakech, Morocco; ^3^Research Laboratory, PCIME, Cadi Ayyad University, 40000 Marrakech, Morocco

## Abstract

*Background*. The Summary of Diabetes Self-Care Activities (SDSCA) is a widely used self-report measure for diabetes self-management. It is an interesting tool for practice and research.* Objectives*. To translate and culturally adapt the SDSCA to the Moroccan context and to assess psychometric properties of the adapted version among type 2 diabetic patients.* Methods*. The Moroccan version was obtained following forward and backward translations. An expert panel issued a final version. The adapted version was administered to patients aged 30 years and older who have type 2 diabetes. Psychometric evaluation consisted of assessing validity through internal consistency (Cronbach's alpha, item-to-scale and interitem correlations) and exploratory factor analysis and reproducibility (test-retest reliability).* Results*. Seventy-five participants were included. Cronbach's alpha ranged from 0.20 (diet) to 0.99 (exercise). Moderate to good interitem and item-to-scale correlations were found. Factor analysis resulted in a Moroccan SDSCA version consisting of 8 items, organized in four subscales that explained 89.6% of the variance: diet, exercise, blood sugar testing, and foot-care. Intraclass correlations ranged from 0.27 to 0.52 for subscales.* Conclusion*. This study provides preliminary evidence for suitability of use of the Moroccan SDSCA among type 2 diabetic patients in order to assess diabetes self-management.

## 1. Introduction

Diabetes represents a leading cause of mortality and morbidity globally. According to recent estimates and projections, specialists talk about a global pandemic of diabetes [1]. In Morocco, available literature reports a prevalence of 6.6% among adults aged 20 years and over [2–4]. Observing and predicting diabetes trends worldwide do draw our attention to this disease and to the challenges raised by its management.

Self-management is a core component of diabetes management given the important role of patient in the context of chronic diseases [5]. The availability of accurate information on self-care activities level alongside with measures of glycemic control should allow care providers to monitor patients' behavior and adjust their interventions to enhance diabetes control. To our knowledge, there is no validated self-care management questionnaire that is used among diabetics in Morocco.

The Summary of Diabetes Self-Care Activities [SDSCA] is a self-report instrument. It measures specific domains of diabetes self-management: diet [general and specific], blood sugar testing, exercise, foot-care, and cigarette smoking [6]. The SDSCA is simple, is easy to complete, and has acceptable psychometric properties [7, 8]. In addition, the instrument is in the public domain and can be used for free. Thus, it is one of diabetes self-management measures that have been largely used. For these reasons, this might be an interesting tool to be used for clinical and research purposes in our context.

Many translations of the SDSCA have been published: Spanish, Turkish, Portuguese, Malay, Italian, and Korean [9–14]. An Arabic version of the SDSCA was recently adapted and validated for Saudi Arabia [15]. Because spoken Arabic in Morocco [Moroccan dialect] is quite different from classical Arabic, it is not appropriate to use the available Arabic version for all Moroccan patients, especially among illiterate people. Thus, it is justified to have a Moroccan adaptation of the instrument.

The aims of this study were to translate and culturally adapt the Summary of Diabetes Self-Care Activities instrument to the Moroccan context and to assess psychometric properties of the adapted version among type 2 diabetic patients.

## 2. Methods

### 2.1. The Summary of Diabetes Self-Care Activities Questionnaire

The SDSCA is a multidimensional instrument used as a direct measure of patient's behavior regarding diabetes self-management. The original questionnaire comprised 14 items and assessed five domains of diabetes management: general diet, specific diet, exercise, medication taking, and blood-glucose testing. Later, items concerning foot-care and cigarette smoking were added. The revised version consists of 11 items that assess diet (general and specific), exercise, blood sugar testing, foot-care, and cigarette smoking [6]. A set of 14 additional items were also proposed. For practicability and ease of use, we decided to adapt the brief version of 11 items.

The first ten items measure the frequency of performing self-management activities for each domain during the last seven days (Table 6). Responses range from 0 to 7 (number of days), with higher scores corresponding to better performances. Item 2 is different from the rest of the items because it inquires about an average week frequency over the last month: “On average, over the past month, how many days per week have you followed your eating plan?” The score of each domain is computed as the mean value of its two constituting items. The last item which concerns smoking over the last seven days has two response modalities: yes or no. Smokers are then requested to report the number of cigarettes smoked on an average day.

### 2.2. Translation and Cultural Adaptation

Permission to adapt the instrument was obtained from the author. The adaptation process was performed according to the forward-backward translation method [16]. Two forward translations from English to Moroccan dialect were independently performed by a physician and a medical student whose mother tongue is Moroccan dialect. A consensual version was obtained during a meeting with the study coordinator. Backward translation was performed by a bilingual physician who was blinded from the original English version.

The expert panel was constituted from a diabetes specialist, a psychologist, an Arabic teacher, two diabetic patients (one of them was a leader in a diabetics' association), an epidemiologist, and a medical trainee. The committee reviewed all versions and managed to reach consensus about discrepancies. A convenience sample of ten patients was recruited for pretest. The final version was then ready for validation without further modifications.

### 2.3. Validation Study

We adopted the design of a longitudinal study. Type 2 diabetic patients aged ≥30 years and treated for diabetes for at least 1 year represented target population. A volunteers' sample was recruited in an association of diabetic patients in Marrakech Region. Patients were approached by a trained interviewer during their usual visits to the association for various activities. Patients with cognitive impairment and those who do not speak Moroccan dialect fluently were excluded due to the impossibility or difficulty to assess the adapted version.

The questionnaire was administered to consenting individuals; additional data were gathered concerning age, gender, literacy, marital status, and professional activity. We also recorded data on disease duration and treatment regimen. Participants were invited to answer the Moroccan SDSCA one month later. To be included for the final analysis, participants should have completed the two administrations of the scale. Participants were asked whether they performed any medical visit during the time interval between the two administrations and whether their treatment regimen was changed.

Statistical analyses were performed using SPSS version 16.0. Data were described using means and standard deviations for quantitative variables and frequencies for qualitative variables. Psychometric analysis concerned the first ten items of the questionnaire. Internal consistency was assessed for each domain and for all items using Cronbach's alpha. Cronbach's alpha value of ≥0.5 was considered acceptable and a value of ≥0.7 was considered good. Item-to-scale correlations between subscale scores and their constituent items were assessed using the Spearman's rank correlation (*r*); correlation was considered acceptable when *r* ≥ 0.4. Interitem correlations were assessed for items within each subscale separately using the Spearman's rank correlation.

Exploratory factor analysis used principal component analysis to extract factors with an eigenvalue criterion of 1. Factors loadings were obtained using Varimax rotation method. Kaiser-Meyer-Olkin measure for sampling adequacy and Bartlett-test for sphericity are presented as well as factors loadings for the items. Test-retest reliability was assessed using intraclass correlation coefficient (ICC) for individual items and subscales. ICC values are presented with confident intervals.

## 3. Results

### 3.1. Cross-Cultural Adaptation

In general, the process of forward and backward translations did not yield many discrepancies. Nonetheless, the experts' committee was challenged by some difficulties and needed to obtain consensus.

The first challenge was to keep in mind that the questionnaire will be administered by interviewers as the questionnaire was not intended for self-administration in our population because of high rates of illiteracy.

Item 2 (general diet) was problematic because it seeks information about an average frequency for week over the last month which was different from the rest of the items which seek information on the frequency over the last week. The study team thought it might be confusing for respondents.

Item 3 inquires about the frequency of consumption of at least 5 servings of fruits and vegetables. We were concerned about the adaptation of this item because family dishes are a common habit in Morocco, which makes it difficult for individuals to quantify their personal intake of any product.

Item 4 introduces the concept of high fat products which was difficult to translate to Moroccan dialect. This concept is not common and thus may not be uniformly understood among the study population.

The expert panel discussed all adapted items and issued a final version that preserved original concepts. It was not clear to what extent the items that appeared somewhat problematic will be understood and pertinent for the target population but the panel decided to keep all items for further analysis (Table 6).

### 3.2. Sample Characteristics

A total of 75 type 2 diabetic patients were recruited for validation study. Their mean age was 56.1 ± 11.6 (range 33–83). Females represented 78% (*n* = 56). A majority of respondents (84%) were illiterate.  Table 1 summarizes patients' characteristics.

Number of years with diabetes ranged from 1 to 29 (mean = 8.1 ± 6.4). The median of diabetes duration was 6 years. Thirty-seven patients were prescribed insulin, which represented 49%. During the time interval between the first and second administrations of the scale, 22 participants reported a medical visit but none of them reported changes of treatment regimen.

### 3.3. Psychometric Properties

Cronbach's alpha coefficient for the 10 items was 0.44. It exceeded 0.9 for general diet and exercise. This coefficient was good for blood sugar testing domain and moderate for foot-care domain. The lowest Cronbach's alpha value was 0.16 for specific diet (Table 2).

Interitem correlation was measured for the five domains using Spearman's rank coefficient. General diet and exercise domains presented correlation over 0.9. Moderate interitem correlations were shown for blood sugar testing and foot-care subscales. Items 3 and 4 were not correlated between them (Table 2). Item-to-scale correlations ranged from 0.58 to 0.99. All items but three (3, 4, and 8) exceeded 0.8 for correlation to their corresponding subscales. Specific diet subscale showed the lowest internal consistency patterns (Table 2).

Factor analysis was performed in two steps. In the first step, the 10 items were included for principal factor analysis. Kaiser-Meyer-Olkin measure for sampling adequacy was 0.5 and the Bartlett's test of sphericity was statistically significant (*p* < 0.001). This analysis loaded 5 factors with eigenvalues greater than 1 that explained 88.3% of total variance (Table 3). All items loaded in their respective subscales except for items 3 and 4. Item 4 loaded in a separate factor and item 3 in the first factor corresponding to “blood sugar testing” subscale.

In the second step, factor analysis was performed after deleting items 3 and 4 (specific diet subscale) due to their weak performance in the first step and to their weak internal consistency. Kaiser-Meyer-Olkin measure for sampling adequacy was 0.47 and the Bartlett's test of sphericity was significant (*p* < 0.001). This analysis loaded 4 factors that explained 89.6% of variance (Table 4). Cronbach's alpha of the 8-item version was 0.48.

Test-retest reliability as measured by ICC was weak (Table 5). We noticed that test-retest reliability was higher for subscales than for individual items. Foot-care subscale showed the lowest ICC. Items of general diet and exercise subscales had similar ICC values, while items of the other subscales had substantially different ICC values.

## 4. Discussion

During the process of translation and cultural adaptation, we faced some challenges that were overcome by the expert panel. The presence of patients in the expert panel was particularly valuable and helped reaching consensus with regard to discrepancies. The most problematic items were 3 and 4 which appear to be related to complex new concepts for the studied population, namely, consumption of high fat foods and servings of fruits and vegetables. Item 4 appeared to be problematic in many previous adaptations [9, 10].

Internal consistency was good for 3 domains: general diet, exercise, and blood sugar testing and moderate for foot-care. Specific diet was the least reliable domain according to Cronbach's alpha coefficient and interitems correlations. This finding is consistent with previous results [6].

Factor analysis showed that most of the items loaded on their original domains, which supports the construct validity of the questionnaire. Furthermore, this analysis resulted in 4 independent factors which confirm the multidimensional character of the measure. Items 3 and 4 did not load on the intended factors. Jalaludin et al. also faced similar challenges with item 4 for the Malay version of the questionnaire adapted for children and adolescents. In the factor analysis, item 4 loaded in the “blood sugar testing” subscale as it did in the first step of factor analysis of our data. In the final Malay version item 4 was replaced by another item from the expanded version [13]. We propose a Moroccan version of the SDSCA consisting of 8 items because items 3 and 4 presented problems during adaptation and showed low psychometric performance later during validation. AlJohani et al. also proposed an adapted Arabic version consisting of 8 items [15].

We found weak correlations for test-retest reliability of the Moroccan SDSCA. Previous data report a mean value of 0.4 for time intervals ranging from 3 to 4 months [8]. Vincent et al. found acceptable to good correlations when they evaluated their Spanish version of the questionnaire [9]. The Arabic version demonstrated excellent test-retest reliability for a 1-week interval [15]. We assumed that one-month interval was long enough to avoid memory bias but short enough to be sure disease patterns did not significantly change. Our findings for test-retest reliability may be explained by maturation bias. The administration of the questionnaire for the first time may have induced a change in self-care patterns among participants. On the other hand, high test-retest reliability reported in other studies may be explained by memory bias when short time intervals are used. Assessing test-retest reliability may be justified during future evaluation of the questionnaire.

Sensitivity to change was not assessed in this study. Responsiveness index ranged from −0.09 to 0.43 [6]. To our knowledge, this feature was not previously assessed in adaptation studies. In addition, we did not assess criterion validity. This property was previously assessed for diet and exercise subscales and coefficients ranged from −0.20 to 0.53 [6]. Criterion validity and sensitivity to change should also be considered in future evaluation of the Moroccan SDSCA.

Further evaluation of psychometric properties of the adapted scale is suitable as it will shed more light on problematic items or dimensions in our culture and allow us to come out with a more accurate set of items. At the same time, we need to try to preserve the core concepts that are explored by the scale so that international and cross-cultural comparisons are made possible. “Specific diet” items proved to be difficult to be understood by participants. We proposed to remove this dimension. Two dimensions need thorough evaluation: blood sugar testing and foot-care. Blood-glucose testing prescription is quite heterogeneous among type 2 diabetics and depends on treatment regimen, glycemic control, and the presence of complications. This may justify assessing psychometric performance of the corresponding items among different patients subgroups. Items related to foot-care appeared to be confusing for participants. Many of them appeared to be surprised by the question about the frequency of checking their feet and some appeared even more surprised by the question about the frequency of checking their shoes. This highlights the need to emphasize self-management in our context and the importance of having validated assessment tools for this care component.

This version was validated among patients mainly of low socioeconomic status, most of whom were illiterate. The presented results may be interesting as they reflect the reality in our context, where it is estimated that almost 1/3 of people aged over 15 years are illiterate [17]. This proportion is even higher among the oldest age groups who suffer from chronic disease such as diabetes. This means that we need tools and methods that are appropriate for illiterate patients. We believe that this version is suitable for literate patients as well but validity and reliability need to be assessed for self-administration, which may be highly desirable when physicians face literate patients.

## 5. Conclusion

The Moroccan version of the Summary of Diabetes Self-Care Activities scale has acceptable validity and reliability. The scale assesses core components of diabetes self-management while it is simple and easy to administer. It may be used in clinics in order to assess reported frequency of self-management activities among patients in our context. Repeating administration of the SDSCA should allow for monitoring patients and identifying areas for behavioral interventions to promote patients' self-management. This study is also a step in providing adapted tools to be used by clinicians and researchers in our context. Such tools are a cornerstone to help advance health care practice and research for diabetes management.

## Figures and Tables

**Table 1:** Sociodemographic characteristics of study participants.

	Number	Percentage (%)
Sex		
Males	19	25
Females	56	75
Literacy status		
Literate	12	16
Illiterate	62	84
Marital status		
Single	6	8
Married	47	63
Widowed	18	24
Divorced	4	5
Professional activity		
Yes	20	27
No	55	73

**Table 2:** Internal consistency of the Moroccan Summary of Diabetes Self-Care Activities measure subscales.

Domains	Items	Item-to-scale correlation^*∗*^	Interitem correlation^*∗*^	Cronbach's alpha
Diet				0.20

General	1 2	0.94 0.99	0.91	0.96

Specific	3 4	0.65 −0.99	0.06 (NS)	0.16

Exercise	5 6	0.94 0.99	0.98	0.99

Blood sugar testing	7 8	0.98 0.58	0.44	0.82

Foot-care	9 10	0.82 0.86	0.46	0.63

All items				0.44

^*∗*^Spearman's rank correlation; all correlations were significant (*p* < 0.001). NS: nonsignificant.

**Table 3:** Results of exploratory factor analysis of the Moroccan Summary of Diabetes Self-Care Activities items (10-item version): factors loadings and explained variance.

Items	Components				
	1	2	3	4	5
1			0.968		
2			0.980		
3	0.745				
4					0.953
5		0.990			
6		0.990			
7	0.893				
8	0.882				
9				0.823	
10				0.872	
Eigenvalues	2.146	2.046	1.945	1.496	1.192
% of variance	21.46	24.47	19.45	14.96	11.92
Cumulative % of variance	21.46	41.92	61.73	76.34	88.25

**Table 4:** Results of exploratory factor analysis of the Moroccan Summary of Diabetes Self-Care Activities items (8-item version: items 3 and 4 deleted): factors loadings and explained variance.

Items	Components			
	1	2	3	4
1		0.979		
2		0.978		
5	0.989			
6	0.991			
7			0.926	
8			0.920	
9				0.853
10				0.854
Eigenvalues	2.048	1.925	1.708	1.485
% of variance	25.60	24.06	21.36	18.56
Cumulative % of variance	25.60	49.67	71.02	89.58

**Table 5:** Test-retest reliability of the Moroccan version of the Summary of Diabetes Self-Care Activities (intraclass correlation coefficient and 95% confidence interval).

Domains	Items	Intraclass correlation	95% confidence interval
General diet		0.45	0.13; 0.65
	1	0.47	0.16; 0.66
	2	0.42	0.08; 0.65

Specific diet		0.47	0.17; 0.67
	3	0.66	0.46; 0.78
	4	0.07	−0.47; 0.41

Exercise		0.39	0.04; 0.62
	5	0.39	0.04; 0.62
	6	0.38	0.03; 0.61

Blood sugar testing		0.52	0.24; 0.70
	7	0.67	0.47; 0.79
	8	0.10	−0.43; 0.43

Foot-care		0.27	−0.15; 0.54
	9	0.01	−0.56; 0.38
	10	0.35	−0.03; 0.59

**Table 6:** The Moroccan Summary of Diabetes Self-Care Activities measure.

	The questions below ask you about your diabetes self-care activities during the past 7 days. If you were sick during the past 7 days, please think back to the last 7 days that you were not sick.	هاد الأسئلة خاصة بالأنشطة لي لازم تدير باش تحمي راسك من السكر فسبعيام لخرة، إلا كنتي مريض فهاد سبعيام لخرة فكر ف آخر سبعيام ماكنتيش فيها مريض

	**Diet **	**الريجيم**

1	How many of the last seven days have you followed a healthful eating plan?	شحال من نهار ف سبع يام لخرة تبعتي برنامج صحي ديال الماكلة (درتي الريجيم)؟

2	On average, over the past month, how many days per week have you followed your eating plan?	ف الشهر لخر، شحال تقريبا من نهار ف سيمانة تبعتي برنامج لماكلة ديالك (الريجيم)؟

3	On how many of the last seven days did you eat five or more servings of fruits and vegetables?	شحال من نهار ف سبع يام لخرة، كليتي 5 وحدات (حبات) أولا كثر من الخضرة اُو الديسير؟

4	On how many of the last seven days did you eat high fat foods such as red meat or full-fat dairy products?	شحال من نهار ف سبع يام لخرة، كليتي ماكلة عالية الدهون/ميدمة (بحال الـلحوم الحمراء اولا منتوجات دهنية عالية الدسم)؟

	**Exercise **	**الرياضة**

5	On how many of the last seven days did you participate in at least 30 minutes of physical activity? (total minutes of continuous activity. including walking).	شحال من نهار ف سبع يام لخرة، درتي على الأقل ف 30 دقيقة ديال النشاط البدني/الحركة؟ (مجموع الدقائق ديال النشاط المستمر بما فيها المشي)‏

6	On how many of the last seven days did you participate in a specific exercise session (such as swimming. walking. biking) other than what you do around the house or as part of your work?	شحال من نهار ف سبع يام لخرة، درتي شي رياضة (بحال السباحة، المشي، ركوب الدراجة...) بلا داكشي لي كادير ف الدر اولا الخدمة؟

	**Blood sugar testing**	**فحص السكر في الدم**

7	On how many of the last seven days did you test your blood sugar?	شحال من نهار ف سبع يام لخرة، درتي تحليلية السكر ديال الصبع؟

8	On how many of the last seven days did you test your blood sugar the number of times recommended by your health care provider?	شحال من نهار ف سبع يام لخرة، عبرتي السكر ف الصبع عدَد لمرات لي كاليك الطبيب؟

	**Foot-care**	**اﻹهتمام بالرجل**

9	On how many of the last seven days did you check your feet?	شحال من نهار ف سبع يام لخرة، قلبتي رجليك؟

10	On how many of the last seven days did you inspect the inside of your shoes?	شحال من نهار ف سبع يام لخرة، قلبتي لداخل ديال سباطك؟
